# Parastomal hernia after robot-assisted cystectomy and ileum conduit: incidence, risk or protective factors and outcomes of minimal-invasive hernia repair-systematic review

**DOI:** 10.1007/s11701-026-03539-7

**Published:** 2026-06-16

**Authors:** Stephan Buse, Julia Neldner, Pawel Rachubinski, Abolfazl Hosseini-Aliabad, Andre Reitz, Axel Haferkamp, Kinan Almansur

**Affiliations:** 1 Urology Department, Alfried Krupp Hospital, Hellweg 100, Essen, 45276 Germany; 2 Clinic and Polyclinic for Urology and Pediatric Urology, University Medical Center of the Johannes Gutenberg University Mainz, Mainz, Germany; 3 Department of Molecular Medicine and Surgery, Section of Urology, Karolinska Institutet, Stockholm, Sweden; 4 KontinenzZentrum AG, Klinik Hirslanden, Zurich, Switzerland

**Keywords:** Robot-assisted cystectomy, Ileum conduit, Parastomal hernia, Minimal-invasive hernia repair

## Abstract

**Background and aims:**

Incidence of and risk factors for parastomal hernia after robot-assisted (RA) cystectomy with ileum conduit (IC) are not well established. The aims of this systematic review were to summarize the literature (1) on the incidence of parastomal hernia after RA cystectomy and IC reconstruction; (2) on risk or protective factors associated with the development of parastomal hernias after RA cystectomy and IC reconstruction; and (3) on outcomes of minimal invasive parastomal hernia repair in patients with IC.

**Methods:**

we conducted literature searches in Medline, EMBASE, and CINHAL up to April 2025 without language restriction. Two independent assessors evaluated eligibility and quality of the included study. Due to high heterogeneity, meta-analysis was not attempted.

**Results:**

out of 368 records, 11 and 2 papers provided information on the incidence of parastomal hernia and on risk factors, respectively. For the outcome of minimal invasive IC hernia repair, 7 studies were included. Most studied presented high risk of bias and the incidence rate varied widely across studies. Similar findings were observed for hernia recurrence after minimal invasive hernia repair.

**Conclusion:**

A relevant proportion of patients may experience parastomal hernia of IC after RA-cystectomy, however incidence figures varied widely. Information on factors influencing the development of parastomal hernia after RA cystectomy is essentially lacking. Finally, the evidence on the outcome of minimal invasive hernia repair in IC patients is very limited both in terms of quantity and of quality. There is urgent need to address the knowledge gaps detected by this systematic review. Cooperation to achieve prospective multicentre designs with adequate sample size and systematic follow-up methods would be crucial factors to generate the high-quality data required to develop evidence-based strategies to prevent parastomal hernias.

## Background

The incidence of parastomal hernias after radical cystectomy and ileum conduit is estimated at 4% to 24% [1]. The need for repair surgery varied from 9 to 75% [1]. Robot-assisted (RA) cystectomy with intracorporal urinary diversion has become standard of care in many institutions, however data on the incidence of parastomal hernia after RA cystectomy with ileum conduit (IC) are limited. In a single-centre cohort of round 400 patients [2], the incidence of parastomal hernias was estimated at 20%. Thereof, 31% of patients were symptomatic and 15% required surgical repair [2]. Another single-centre study (*n* = 284 RA cystectomies) reported that 43% of patients developed parastomal hernia of IC during 2-years follow-up [3]. Therefore, there is relevant uncertainty regarding the incidence of parastomal hernia of IC after RA-cystectomy. Similarly, factors influencing the incidence of parastomal hernia after RA cystectomy are not established [2–4]. Finally, the outcomes of minimal-invasive parastomal hernia repair in IC patients remain rarely described [5–7].

The aim of this systematic review was to summarize the literature (1) on the incidence of parastomal hernia after RA cystectomy and IC reconstruction; (2) on risk or protective factors associated with the development of parastomal hernias after RA cystectomy and IC reconstruction; and (3) on outcomes of minimal invasive parastomal hernia repair in patients with IC.

## Methods

We conducted a systematic review on the incidence of and on risk or protective factors for parastomal hernia after RA cystectomy and ileum conduit (IC) in adults. A second systematic literature search focused on outcomes reported for minimal-invasive (RA or conventional laparoscopy) repair of parastomal hernias of IC. Data were extracted from published literature so that ethical board approval was not required. This report adheres to the PRISMA statement.

### Literature search

A literature search in Medline, EMBASE, and CINHAL [8] was conducted from 1 January 1946 − 11 April 2025. The databases were chosen based on expected high subject coverage, specifically on the incidence of parastomal hernia. The search was augmented by manual search. We did not apply any language restrictions. The search was developed in Medline and adapted to the other search engines. Key components included “parastomal”, “hernia”, “ileum OR ileal AND conduit“, and “urinary diversion”. In order to increase sensitivity, i.e., to avoid missing reports including robot-assisted and open procedure, we did not include the term robot-assisted in the search and limited the inclusion to papers with robot-assisted procedures during eligibility assessment (see exclusion criteria). Studies on RA cystectomy with intra- and extra-corporal ileum conduit were included. Studies were included independent of their approach to parastomal hernia detection (systematic diagnostic work-up or clinical manifest hernias, see below). The other search (aim 3) included “minimal-invasive OR laparoscopic OR robot-assisted”, “hernia repair”, “(ileum OR ileal AND conduit) OR urinary diversion”. As long as the hernia repair was minimal-invasive, studies were included independent of the surgical approach to the initial cystectomy and IC.

The search hits were exported to a Rayyan© database for screening. Eligibility was assessed by 2 independent reviewers. Disagreement was solved by discussion with involvement of a third senior assessor (SB).

The following studies were excluded:

- Experimental studies (animal or material/tissue studies).
- Narrative reviews or letters to the editor (non-research letters).
- Children (< 18 years).
- Cystectomy was not robot-assisted or in studies reporting on various routes for radical cystectomy, no separate findings for robot-assisted procedure were available/provided.
- Parastomal hernia repair was not minimal-invasive or in studies reporting on mixed populations (minimal-invasive and open or different types of hernia) no specific data available.
- Case reports or series on minimal-invasive hernia repair reporting on less than 10 patients.
- Outcomes of interest not reported.

### Endpoints

The development of any parastomal hernia (symptomatic, clinically manifest, or radiologically detected- as defined by the single studies) after RA cystectomy was the primary endpoint. Secondary endpoints were symptomatic parastomal hernia and parastomal hernia requiring surgical repair.

For aim 3 (outcome of minimal-invasive parastomal hernia repair) the primary endpoint was parastomal hernia recurrence. However, any clinical endpoint (complications, mortality, quality of life, …) and surgical benchmarks as available in the included studies were considered.

### Risk or protective factors for parastomal hernia

Any risk or protective factor assessed, e.g., demographic data, details of surgical technique, comorbidities, and postoperative complications as explored and reported in the included studies was considered.

### Data extraction and risk of bias assessment

Data were extracted into a predefined template and included the following information on methodology: study design, eligibility criteria, period of enrolment, details of initial procedures, methods for parastomal hernia detection, follow-up duration and approach to follow-up. Study results extracted included sample size, follow-up completeness, if applicable mean/median follow-up duration, baseline characteristics (age, body mass index, indication for RA cystectomy, comorbidities as far as reported), surgery duration, intra- and postoperative complications, and risk or protective factors for parastomal hernia assessed and their effect size (as far as reported). For studies included for aim 3, the following information was extracted: study design, eligibility criteria, period of enrolment, type of minimal-invasive approach, planned primary endpoint, planned follow-up duration, sample size, parastomal hernia severity and classification tool at baseline, age (mean/median), BMI (mean/median), ASA class, surgery duration, conversion rate, blood loss, transfusion rate, intraoperative complication rate, postoperative complications (including time horizon) and length of stay. For hernia recurrence, we collected approach to recurrence detection, follow-up duration, loss of follow-up, and incidence of parastomal hernia recurrence.

Data was extracted by trained extractors. Authors of relevant studies were contacted to obtain missing information. Similarly, authors of manuscripts reporting on various approaches to cystectomy, i.e., mixed population with open and RA-procedures were contacted for data specific to RA cystectomy. Specific data to minimal invasive hernia repair in ileum conduit were also requested for studies reporting on mixed populations, e.g., reporting on open and minimal-invasive hernia repair or on minimal-invasive hernia repair for different types of parastomal hernia (e.g., colostomy, ileostomy and ileum conduit). If authors did not respond after a third reminder to provide the requested information, the data was considered as not available and if no separate findings specific to RA cystectomy or minimal invasive parastomal hernia repair for ileum conduit were provided, the study was excluded.

Methodological quality of the included cohort was assessed using the Newcastle-Ottawa Scale for cohort, JBI critical appraisal tool for case series [9], and Cochrane Risk Of Bias (ROB) 2 tool for Randomized Control Trial (RCT). The outcome for ROB assessment using ROB 2 tool was parastomal hernia development. Of note, the RCTs reported on open and RA procedures and risk of bias was assessed for the whole trial (not specifically for RA procedures).

### Analyses

In consideration of the expected heterogeneous duration of follow-up and methods of assessment of parastomal hernia, we primarily planned to summarize the incidence data descriptively while maintaining an option for meta-analytic pooling. The same applied to minimal-invasive hernia repair. The review of the available data confirmed the expected heterogeneity and a meta-analysis was not attempted. A meta-analytic approach for risk or protective factors and for outcome was never planned.

## Results

**Fig. 1:**
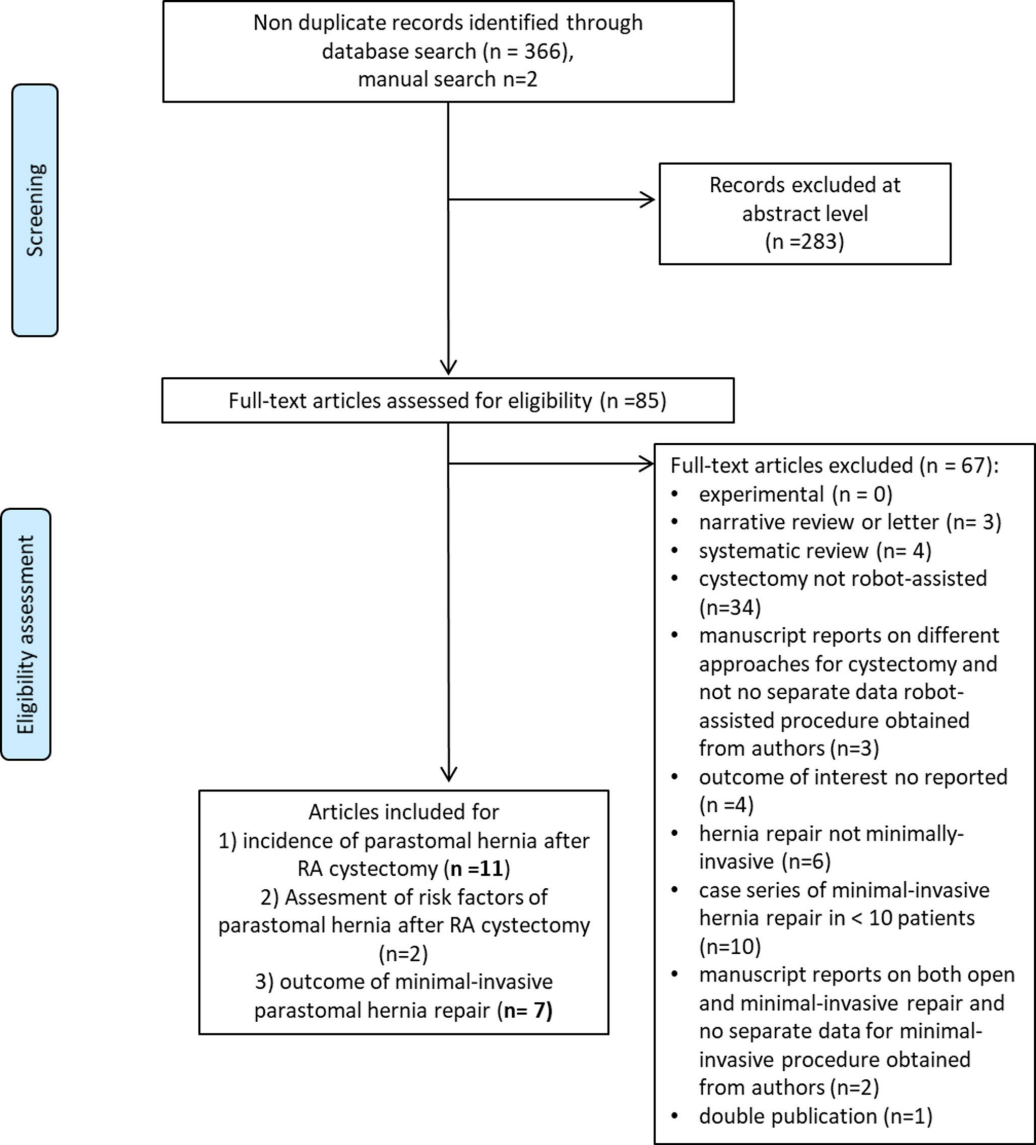
PRISM Flow chart of study selection

After duplicate removal, 368 records were identified. Thereof, 283 were excluded during title and abstract screening. After full-text eligibility assessment, 11 papers [2–4, 10–17] were included for the assessment of the incidence of parastomal hernia, thereof 2 [2, 11] provided information on risk or protective factors for parastomal hernia. For the outcome of minimal invasive parastomal hernia repair in ileum conduit, 7 studies [5–7, 18–21] were included. Reasons for exclusion in the full-text eligibility assessment are reported in the PRISMA flow chart (Fig. 1).

### Quality assessment

Of the 11 studies for the assessment of the incidence of parastomal hernia after RA-cystectomy with ileum conduit, 9 (81.8%) were retrospective single-centre case series and 2 (18.2%) RCTs. Of the 9 case series, 4 (44.4%) presented high-risk of bias due to selection bias arising from relevant proportions of patients excluded due to missing data and due to high attrition. For the other 5 studies, risk of bias was unclear since information on missing data and on loss of follow-up was not reported. For both RCT, risk of bias was low for all domains of the ROB2 tool (of note the assessment referred to parastomal hernia after open or RA procedures [11]and open, laparoscopic and RA procedures [17], respectively since both RCT included different surgical techniques).

Of the 7 studies reporting on minimal-invasive hernia repair for parastomal hernia in ileum conduit patients, 5 (71.4%) were retrospective single-centre case series with high or unclear risk of bias mainly due to selection and classification bias resulting from missing data and loss of follow-up and 2 studies, a retrospective single-centre [19] and a retrospective multicentre cohort [6] presented low risk of bias.

### Incidence of parastomal hernia after RA cystectomy and ileum conduit

Eleven studies reported on the incidence of parastomal hernia in a total of 920 patients. The sample size ranged between 9 and 383 patients, the follow-up duration from 17 days to 45 months (median or mean depending on the study). As far as reported, the samples consisted mainly of men. The mean or median age ranged from 62 to 75 years, mean or median BMI from 22 to 30 kg/m2. Parastomal hernia was observed in 0 to 44% of patients after RA-cystectomy with IC diversion. Of note, in the 2 largest studies [2, 3], parastomal hernia was reported in 19.6% [2] and 43% of patients [3], respectively.

In 4 studies with a total of 490 patients [2, 11, 12, 17] symptomatic hernias were reported and affected 0 to 9.5% of patients. Reoperation for parastomal hernia was necessary in 0 to 16.6% patients (assessed in 5 studies [2, 10–12, 17] with a total of 518 patients).

The proportion of intracorporal IC creation ranged from 0 to 100%. Data were not sufficient to assess the impact of intra- vs. extra-corporal technique on parastomal hernia incidence.

Table 1 reports the details of the studies included for the assessment of incidence of parastomal hernia.

**Table 1:** Characteristics and findings of the studies reporting on the incidence of parastomal hernia after RA cystectomy and IC

Author	Study design	Period of enrolment	*N*=	M: F	Age (mean/median)	BMI (mean/median)	Proportion of Intracorporal ileum conduit technique	Follow-up duration (mean/median	*N* (%) parastomal hernia	Mean/median time to parastomal hernia	*N* (%) symptomatic parastomal hernia	*N* (%) parastomal hernia requiring surgery repair
**Miyake M**,** Asian J Endosc Surg 2025** [14]	single-centre retrospective case series	2019–2022	27	24?M, 4 F	75y (71–79)	22.2 (19.6–24.3)	100%	median 20 mt	1 (3.7%)	11 mt	not reported	not reported
**Lone Z**,** BJU Int 2024** [3]	single-centre retrospective case series	2010–2020	284	not reported for RARC only	70y (whole cohort)	median BMI 26 in no PH, 30 in pat with PH (whole cohort)	59%	median follow-up 45 mt in no PH and 43 mt in pat with PH (whole cohort)	125 (44.0%)	“125 hernia at 2 years”	not reported	not reported for RARC only
**Faria EF**,** International Braz J Urol 2024 **[12]	single-centre retrospective case series	2018–2023	28	not reported for IC only	65y (46–88)	26.8 (17–40)	100%	90 days	0	90 days	0	0
**Djaladat H**,** J Urol 2024 **[11]	single-centre RCT	2015–2021	73	58 M, 15 F	74y	26.7	100%	20.4 mt (IQR 8–25)	18 (24.6%)	20.4 months (IQR 8–25)	7 (9.6%)	2 (2.7%)
**Carlton CE**,** Urology 2024** [10]	single-centre retrospective case series	2011–2021	24	0 M, 24 F	75y (range 53–87)	26 (23–30)	not reported	14 mt (IQR 5–28)	7 (29.2%)	1–3 years	not reported	4 (16.7%)
**Zhou Z**,** Cell Rep med 2024 **[17]	single-centre RCT	2019–2020	10	not reported for RARC only	62y ± 11 (whole cohort)	mean 22.9 (whole cohort)	not reported	31 mt (12–35) (whole cohort)	2 (20%)	not applicable	0	0
**Rezaee ME**,** Can J Urol 2020** [4]	single-centre retrospective case series	2009–2018	9	not reported for RARC only	71y (whole cohort)	mean 30.1 (whole cohort)	not reported	3.4 years (whole cohort)	2 (22.2%)	not reported	not reported	not reported
**Tenzel PL**,** Hernia 2018 **[16]	single-centre retrospective case series	2010–2017	38	28 M; 10 F	68y	not reported	0%	21 mt in patients with and 11 mt without mesh	1 (2.6%)	not reported	not reported	not reported
**Hussein AA**,** J Urol 2018** [2]	single-centre retrospective case series	2005–2016	383	not reported	not reported	pat with vs. without PSH BMI 30 vs. 28 kg/m2 (*p* < 0.02) (whole cohort)	100% after 2009	18 mt (IQR 7–35)	75 (19.6%)	13 months (IQR 9–22)	23 (6.0%)	11 (2.9%)
**Huynh D**,** J Robot Surg 2017** [13]	single-centre retrospective case series	2013–2015	15	12 M, 3 F	72y (58–90)	mean 29 (23–43)	0%	17 d (10–40)	0	not applicable	0	0
**Pruthi RS**,** Urology 2008 **[15]	single-centre retrospective case series	2006–2007.	29	not reported for IC subgroup	64y (whole cohort)	not reported	0%	13 mt (range 2–24) (whole cohort)	3 (10.3%)	not reported	not reported	not reported

### Risk or protective factors for parastomal hernia after RA cystectomy with ileum conduit

For 2 studies (1 RCT and 1 large retrospective case series) [2, 11] with a total of 93 hernias in 456 patients, data specific for parastomal hernia in IC after RA cystectomy were available to assess risk or protective factors. In patients submitted to RA cystectomy, the prophylactic use of mesh [11] was not protective. Hussein et al. [2] reported longer surgery duration (adjusted odds ratio [OR]1.25, 95% CI 1.06–1.46), fascial defect size ≥ 30 mm (adj OR 5.23, 95% CI 2.32–11.80), and postoperative eGFR less than 60 ml per minute (adj OR 2.17, 95% CI 1.23–3.90) as significant predictive factors for parastomal hernia development.

### Outcome after minimal-invasive parastomal hernia repair after RA cystectomy and ileum conduit

The 7 studies on the outcome after minimal invasive parastomal hernia repair included a total of 137 patients, thereof 25 RA parastomal hernia repairs. Sample size ranged from 10 to 35 patients. Only 2 studies [5, 7] prespecified planned follow-up durations for hernia recurrence. Table 2 reports the characteristics of the studies addressing outcome after minimal invasive parastomal hernia repair and their findings.

**Table 2:** Characteristics and findings of studies reporting on minimal invasive parastomal hernia repair in IC patients

Author	Study design	Period of enrolment	Minimal invasive procedure	Sample size	M: F	Age (mean/median)	BMI (mean/median)	Parastomal hernia classification (European Hernia Society)	Surgery duration (min)	Intraop complications	Postop complication rate	Length of stay	Follow-up duration for hernia recurrence (months)	Incidence of parastomal hernia recurrence
Violante, Hernia 2024 [7]	single-centre retrospective case series	2021–2023	robot-assisted	15	7 M, 8 F	69.1 ± 10.2	31.6 ± 6.2	5x class I, 5x class II, 4x class III, 1x class IV	249 ± 63	not reported	2x ileus, 1x abdominal collections, 1 exploratory laparoscopy	3.6d ± 1.9	15.2 ± 7.6	0/15
Suwa, Journal of the anus, rectum and colon 2024 [21]	single-centre retrospective case series	2012–2023	laparoscopic	10	6 M, 4 F	72 (48–84)	24 (22–32)	5x class I, 3x class II, 2x class 4	223 (120–423]	1 (10%) bowel lesion	1 × (10%) ureteral obstruction	9 d (6–14)	52 (20–104)	0
Fu, BMC Surg 2024 [18]	single-centre retrospective case series	2013–2022	laparoscopic	35	20 M, 10 F	69 (64.5–71)	25.1 (23.3–27.4)	16x class I, 13x class II, 5x class III, 1x class IV	90 (65–120)	3 (8.6%) bowel lesion	1x ileus; 2x peristomal abscess	5 d (4–7)	32 (25.5–38)	3 (8.6) after a median of 14 mt
Goffioul Hernia 2024 [19]	single-centre retrospective case series	2014–2020	laparoscopic	16	9 M, 7 F	67 (65–75)	27,60	6x class I, 5x class II, 4x class III, 1x class IV	76 (58–105)	2 (12.5%) conversion	2x CDC 3a, 1x CDC 3b	3 d (3–4.25)	30 (17–35)	2/16
Dewulf, Langenbeck’s Arch Surg 2022 [5]	single-centre retrospective case series	2016–2020	robot-assisted and laparoscopic	15	10 M, 5 F	75 (73–77)	28.7 (25.7–34.2)	not reported	197 (132–260)	not reported	1x CDC 2, 2x CDC class 3	5 d (3–9)	366 d (241–1286)	1 of 15? (unclear loss of follow-up)
Laycock, Hernia 2022 [20]	single-centre retrospective case series	2008–2019	laparoscopic	27	16 M, 11 F	median age 70 years (mean 67.5)	median 29.6, mean 29.7	not reported	mean 118	not reported	4x CDC 2	median 2 d, mean 3.6d	median 518 days, mean 750	2 (8.7%)
Mäkäräinen-Uhlbäck, BMC Surg 2021 [6]	multicentre retrospective case series	2007–2017	laparoscopic	19	not reported only for minimal invasive	not reported only for laparoscopic	not reported only for laparoscopic	not reported	not reported only for laparoscopic	not reported	1x bleeding, 1x infection (not SSI)	4.8d ± 3.1 (range 2–15)	38+/−26 months (range 1–100)	4 of 19

## Discussion

The main findings of this systematic review are that (1) a relevant proportion of patients may experience parastomal hernia of IC after RA-cystectomy. However, incidence figures varied widely (0 to 43%) and evidence was frequently affected by selection bias, so that the incidence for parastomal hernia of IC after RA cystectomy remains unestablished; (2) information on factors influencing the development of parastomal hernia after RA cystectomy is essentially lacking; (3) the evidence on the outcome of minimal invasive hernia repair in IC patients is very limited both in terms of quantity and of quality.

### Comparison with previous studies

Narang et al. [1] conducted a systematic literature review on the incidence of parastomal hernia of ileum conduit after cystectomy by any surgical technique. Out of 63 hits, they included 12 retrospective case series published between 1989 and 2016. In spite of varying or unclear follow-up duration, they reported a pooled incidence at 17.9% for parastomal hernia development. In the present study, we conducted an extensive search (368 hits) in April 2025 and we focused on robot-assisted cystectomies. Due to heterogenous follow-up durations, we avoided pooling. In the 2 larger samples, 19.6% [2] and 43% [3] of patients after robot-assisted cystectomy and IC creation, respectively, suffered parastomal hernia. In 2020, Feng et al. [22] conducted a systematic literature search and included 15 studies reporting on parastomal hernia after radical cystectomy by any approach. They reported pooled incidences of radiological parastomal hernia of 14% at 1 year and of 26% at 2 years, respectively. However, as in the present review focused specifically on RA cystectomy, incidence of parastomal hernia varied widely between studies. Reasons for the high heterogeneity of the estimated incidence of parastomal hernia after RA cystectomy may include methodological issues, e.g., different follow-up durations, different follow-up approaches, and different definitions (radiological vs. clinically manifest). However, the wide diverging incidence reported in the literature may also arise from the single centre design of most studies, since which surgeon conducted the procedure appears to have a major impact on the incidence of parastomal hernia [4].

With regard to risk factors for parastomal hernia, our findings confirm for RA cystectomy what was described for open cystectomy: the various studies addressed a large number of different risk factors, often without being adequately powered for such analyses, so that robust conclusions in this regard are not possible. In terms of the prophylactic use of mesh to prevent parastomal hernia during IC creation after cystectomy by any surgical technique, the pooled estimates of 3 RCTs suggested a protective effect, albeit with large confidence intervals (OR 0.49 [95%CI 0.25–0.97]) [23].

In addition to summarizing the evidence on the incidence of parastomal hernia and its risk factors with a focus on RA procedures, the current standard of care in many institutions, we expanded the perspective of previous systematic reviews by the consideration of outcomes after minimal-invasive parastomal hernia repair and the conduction of a corresponding systematic search. The body of evidence on this topic is severely limited. At the current stage, evidence is so limited that adequate patient counselling in term of outcome for minimal-invasive parastomal hernia repair may be considered not feasible.

### Knowledge gaps and future research

This systematic review uncovers the many knowledge gaps related to parastomal hernia of IC after robot-assisted cystectomy: the incidence of parastomal hernia remains unquantified, risk and protective factors are essentially unknown. Finally, the outcomes, surgical benchmarks as well as recurrence, of parastomal hernia repair is insufficiently described. This information however is essential to inform patients on theirs parastomal risk and crucial to design interventions to prevent the development of parastomal hernia. To generate the high-quality data required to address the current knowledge gaps, the conduction of multicentre, prospective studies with uniform definitions and assessments methods, and with adequate follow-up duration, as well as approaches to minimize attrition is required.

### Strengths and limitations

Strength of our approach include an extensive literature search in 3 databases with high expected coverage of the topic, study selection according to predefined criteria by 2 assessors, and a focus not only on the incidence of parastomal hernia after RA cystectomy with IC but also on risk or protective factors for it. Further, this review summarizes also the outcomes of minimal invasive hernia repair, therefore addressing the whole trajectory of patients suffering a parastomal hernia after IC creation.

We are aware of the following limitations: first, follow-up durations in the included studies were highly heterogeneous, thus preventing the calculation of pooled estimates for the incidence of parastomal hernia after RA cystectomy and IC. Second, while efforts were undertaken to obtain information specific to RA procedures, some papers had to be excluded since the necessary information was not provided. Finally, we did not search clinicaltrials.gov for ongoing studies. This decision was driven by the focus on incidence and risk factors that are mostly generated in observational studies and we assumed that observational studies might be less well represented than clinical trials in that repository. Also, we aimed for completed studies with available results.

## Conclusion

A relevant proportion of patients may experience parastomal hernia of IC after RA-cystectomy, however incidence figures varied widely across studies and evidence was frequently affected by selection bias. Information on factors influencing the development of parastomal hernia after RA cystectomy is essentially lacking. Finally, the evidence on the outcome of minimal invasive hernia repair in IC patients is very limited both in terms of quantity and of quality. As such this systematic literature search discloses major knowledge gaps with regard to parastomal hernia after RA cystectomy with IC and its minimal-invasive repair. To develop strategies to prevent hernia development to offer guidance to surgeons and for patients´ counselling, there is an urgent need to address these knowledge gaps. Cooperation to achieve prospective multicentre designs with adequate sample size, systematic follow-up methods, and efforts to limits attrition would be crucial factors to generate the high-quality evidence that is currently missing.

## Data Availability

The datasets used in the current systematic review are available from the corresponding author on reasonable written request.
